# Chemical Compositions and Anti-Skin-Ageing Activities of *Origanum vulgare* L. Essential Oil from Tropical and Mediterranean Region

**DOI:** 10.3390/molecules25051101

**Published:** 2020-03-01

**Authors:** Natnaree Laothaweerungsawat, Jakkapan Sirithunyalug, Wantida Chaiyana

**Affiliations:** 1 Master’s Degree Program in Cosmetic Science, Faculty of Pharmacy, Chiang Mai University, Chiang Mai 50200, Thailand; natnaree.la@gmail.com; 2 Department of Pharmaceutical Sciences, Faculty of Pharmacy, Chiang Mai University, Chiang Mai 50200, Thailand; jakkapan.s@cmu.ac.th; 3 Research Center of Pharmaceutical Nanotechnology, Chiang Mai University, Chiang Mai 50200, Thailand

**Keywords:** *Origanum vulgare*, carvacrol, gas chromatography–mass spectrometry, antioxidant, anti-skin-ageing, collagenase, elastase, hyaluronidase

## Abstract

*Origanum vulgare* L. has been used as a culinary ingredient worldwide. This study revealed the cosmeceutical potential of *O. vulgare* essential oil as a skin-ageing retardant. The *O. vulgare* essential oil from a highland area of a tropical country (HO), obtained by hydrodistillation was investigated and compared to a commercial oil from the Mediterranean region (CO). Their chemical compositions were investigated by gas chromatography–mass spectrometry. Antioxidant activities were investigated by ferric reducing antioxidant power, 1,1-diphenyl-2-picrylhydrazyl, and ferric thiocyanate assay. Anti-skin-ageing activities were determined by means of collagenase, elastase, and hyaluronidase inhibition. Carvacrol was the major component in both oils, but a higher amount was detected in HO (79.5%) than CO (64.6%). HO possessed comparable radical scavenging activity to CO (IC_50_ = 1.8 ± 0.8 mg/mL) but significantly higher lipid peroxidation inhibition (38.0 ± 0.8%). Carvacrol was remarked as the major compound responsible for the reducing power of both oils. Interestingly, HO possessed significant superior anti-skin-ageing activity than ascorbic acid (*P* < 0.01), with inhibition against collagenase, elastase, and hyaluronidase of 92.0 ± 9.7%, 53.1 ± 13.3%, and 16.7 ± 0.3%, at the concentration of 67, 25, and 4 µg/mL, respectively. Since HO possessed comparable anti-hyaluronidase activity to CO and superior anti-collagenase and anti-elastase (*P* < 0.01), HO was suggested to be used as a natural skin-ageing retardant in a cosmetic industry.

## 1. Introduction

*Origanum vulgare* L., which belongs to the family Lamiaceae, is native to the highland area of the Mediterranean region of Europe and Asia [1]. Turkey, which bridges the continents of Europe and Asia, is the biggest exporter of the *O. vulgare* herb and the derived essential oil to world markets [2]. Tropical countries need to import *O. vulgare* from overseas because of inappropriate cultivation conditions. However, *O. vulgare* is currently cultivated in the highland area of some tropical countries, such as Thailand. Nowadays, the Thai Royal Project Foundation encourages people in the highland area (higher than 1000 m above sea level) to cultivate this winter plant instead of shifting cultivation to another area and causing further deforestation [3,4]. Therefore, cultivation of economic crops in the highland area would reduce deforestation and increase the income of highland people. Although locally cultivated *O. vulgare* is now available in Thailand, it is not popular since there was no helpful data about this plant. Therefore, investigation of the biological activities of *O. vulgare* from Thailand would be an interesting research project, to promote new applications of *O. vulgare* and, in turn, encourage local highland people to cultivate *O. vulgare*.

*O. vulgare* has been used as a spice and in ethnomedicine as a stimulant, tonic, carminative, and diaphoretic since ancient times [5,6]. *O. vulgare* has also been reported to have antimicrobial, antifungal, antiviral, analgesic, antioxidant, and anti-inflammatory activities [7,8,9,10]. Additionally, the derived essential oil has been reported as a natural skin penetration enhancer for transdermal drug delivery [11]. Therefore, it has potential for topical application, especially for anti-skin-ageing treatment, due to the previously reported antioxidant and anti-inflammatory activities which protect against the consequences of free radical damage by various endogenous reactive oxygen species (ROS) that are directly associated with ageing and skin ageing [12,13]. ROS, which damage the extracellular matrix in the skin layer, can be stopped by the scavenging activity of antioxidant compounds [14,15]. Normally, the human body has self-protection mechanisms against these ROS, including superoxide dismutase, metallothionein, and melanin. However, once oxidative stress overpowers the defense mechanisms of the skin, damage can occur. Therefore, supporting the skin defense mechanism with exogenous antioxidant compounds would reduce the oxidative process in the body and reduce skin ageing [16,17]. Additionally, the flattening of the dermal–epidermal junction and extracellular matrix atrophy caused by reducing levels of collagen, elastin, natural moisturizing factor, and other evident biological features of skin ageing [13,18]. However, the biological activities related to skin-ageing retardation of *O. vulgare* have not been reported and there were few investigations on *O. vulgare* from tropical regions.

Therefore, the present study was the first to report anti-skin-ageing activity of *O. vulgare* essential oil from a tropical region (Thailand). Inhibitory activities against collagenase, elastase, and hyaluronidase were investigated. In addition, the essential oil of the highland area was compared between a tropical (Thailand) and a Mediterranean (Spain) region.

## 2. Materials and Methods

### 2.1. Plant Materials

Whole plants of *O. vulgare* were obtained from the highland area in Mae Chaem district, Chiang Mai, Thailand, during January 2018. These plant materials were cultivated by the Royal Project Foundation, Thailand. They were authenticated as herbarium specimen number 023235 and deposited at the official Herbarium at the Faculty of Pharmacy, Chiang Mai University, Thailand. The fresh plants were washed and cut into small pieces and used for hydrodistillation.

### 2.2. Chemical Materials

Commercial *O. vulgare* essential oil (CO) was purchased from Botanicessence (Product of Spain) (Bangkok, Thailand). Carvacrol, α-tocopherol, 2,2′-diphenyl-1-picrylhydrazyl (DPPH), 2,4,6, tripyridyl-s-triazine (TPTZ), hydrochloric acid (HCl), acetic acid (CH_3_COOH), linoleic acid, hyaluronidase from bovine testes, sodium chloride (NaCl), collagenase from Clostridium histolyticum, *N*-[3-(2-furyl)acryloyl]-Leu-Gly-Pro-Ala (FALGPA), elastase from porcine pancreas, *N*-succinyl-Ala-Ala-Ala-p-nitroanilide (AAAVPN), sodium phosphate monobasic dihydrate (NaH_2_PO_4_.2H_2_O), and sodium phosphate dibasic dihydrate (Na_2_HPO_4_.2H_2_O) were purchased from Sigma-Aldrich (St. Louis, MO, USA); α-Ascorbic acid was purchased from Asia Pacific Specialty Chemicals Limited (New South Wales, Australia). Anhydrous sodium sulfate (Na_2_SO_4_), ferric chloride hexahydrate (FeCl_3_.6H_2_O), ferrous sulphate heptahydrate (FeSO_4_.7H_2_O), and ammonium thiocyanate (NH_4_SCN) were purchased from Loba Chemie (Boisar, Tarapur, India). Bovine serum albumin was purchased from Merck (Darmstadt, Germany). Tricine was purchased from Bio-Rad Laboratories (Richmond, CA, USA). Sodium acetate trihydrate (CH_3_COONa.3H_2_O), calcium chloride dihydrate (CaCl_2_.2H_2_O), sodium hydroxide (NaOH), dimethyl sulfoxide (DMSO), dichloromethane, methanol, and ethanol were analytical grade and purchased from RCI Labscan Co., Ltd. (Bangkok, Thailand).

### 2.3. Extraction of Essential Oils by Hydrodistillation

Highland *O. vulgare* was subjected to hydrodistillation for 2 h, to extract essential oil (HO), and the essential oil was then kept in a glass bottle after cooling down to the room temperature. The residual water was then removed, using anhydrous sodium sulfate. The yield of essential oil was calculated by using the following equation:

%Yield = (a/b) × 100,(1)

where a is a volume of the essential oil, and b is the weight of fresh plant materials used in the hydrodistillation. The essential oil was stored at 4 °C, in a light-protected container, until further use.

### 2.4. Characterization of *O. vulgare* Essential Oils

The characteristics of HO and CO, including physical appearance, color, and scent, were investigated by organoleptic inspections. Relative density of each oil was also investigated, using a pycnometer. Refractive index was determined by using a refractometer (Altago Co., Ltd., Tokyo, Japan).

### 2.5. Chemical Compositions Determination of *O. vulgare* Essential oils by Gas Chromatography–Mass Spectrometry (GC–MS)

The chemical compositions of HO and CO were investigated by Agilent 6890 GC-MS (AgilentTechnologies, CA, USA), using 30.0 m × 250 mm i.d., 0.25-mm film thickness fused-silica HP-5 ms (HewlettPackard, CA, USA), and hydrogen gas was used as the carrier (mobile phase). The flow rate of the carrier gas was set at 1 mL/min, and the injection temperature was set at 260 °C. The analyses were performed by using a temperature gradient. In particular, column initial temperature was set at 100 °C, which was held for 3 min. Then, the column temperature was raised at 3 °C/min to reach the final temperature of 280 °C, which was maintained for 3 min. The resulting chromatogram was used to identify the chemical compositions of each essential oil by using the differential of the retention time compared with Wiley, NIST, and NBS libraries. Kovats index (KI) was calculated by using the following equation:

KI = 100 × [(tx − tn) / (tn + 1 − tn)],(2)

where KI is the Kovat index; tn, tn+1, and tx are the retention time (in minute) of the two *n*-alkanes containing n, n + 1 carbons, and the compounds of interest, respectively [19].

### 2.6. Determination of Anti-Wrinkle Activities of Essential Oils

The anti-skin-ageing activities of HO, CO, and their major components were investigated by means of antioxidant, anti-hyaluronidase, anti-collagenase, and anti-elastase activity assays.

#### 2.6.1. Determination of Antioxidant Activities

##### 1-Diphenyl-2-picrylhydrazyl (DPPH) Assay

HO, CO, and their major components were investigated for DPPH^●^ scavenging activity [20,21]. Briefly, 180 µL of 167 µM DPPH^●^ solution was mixed with 20 µL of various concentrations of sample in a 96-well (flat) microplate. The mixture was allowed to rest for 30 min, at room temperature, in the dark. The absorbance of the resulting solution was measured at 520 nm by a microplate reader (BMG Labtech, Offenburg, Germany). All measurements were performed in triplicate. The DPPH^●^ inhibition was calculated by using the following equation:

% Inhibition = [1 − (a/b)] × 100,(3)

where a is the absorbance of DPPH^●^ solution and sample solution, and b is the absorbance of DPPH^●^ solution and ethanol. IC_50_ of each sample was calculated by using the program GraphPad Prism Version 2.01 (GraphPad Software, San Diego, USA). Ascorbic acid was used as a positive control.

##### Ferric Reducing/Antioxidant Power (FRAP) Assay

HO, CO, and their major components were investigated for ferric-reducing power by using the FRAP assay [21]. Briefly, freshly prepared FRAP solution was obtained by mixing together 0.3 M acetate buffer (pH 3.6), 10 mM TPTZ solution in 40 mM HCl, and 20 mM ferric chloride solution in the ratio of 10:1:1. After that, 180 μL of FRAP solution was mixed with 20 μL of each sample. Various concentrations of ferrous sulfate (FeSO_4_) solutions were used for calibration curve construction. The ferric-reducing ability of each sample was measured, using a microplate reader (BMG Labtech, Offenburg, Germany), after 5 min of incubation, at an absorbance of 595 nm. The results were reported as equivalent capacity (EC_1_), indicating Ferric Reducing/Antioxidant Power the ability to reduce ferric ions, expressed as mM FeSO_4_ equivalents per mg of the sample. Each experiment was done in triplicate. Ascorbic acid was used as a positive control.

##### Lipid Peroxidation by Ferric Thiocyanate Method (FTC) Method

HO, CO, and their major components were investigated for inhibition against lipid peroxidation using the FTC assay [21]. Briefly, 50 μL of 50% linoleic acid in DMSO was added into the sample solution. The reaction was initiated by the addition of 50 μL of 10% NH_4_SCN solution and 50 μL of 2 mM FeCl_2_ solution. The mixture was incubated at 37 ± 2 °C for 1 h. During the auto-oxidation of linoleic acid, peroxides will be formed that lead to the oxidation of Fe^2+^ to Fe^3+^. The Fe^3+^ ions formed a complex with thiocyanate that can be detected at 500 nm, using a multimode detector (BMG Labtech, Offenburg, Germany). The mixture without sample was used as a negative control. All experiments were performed in triplicate. The inhibition of lipid peroxidation of linoleic acid was calculated by using the following equation:

% Inhibition = [1 − (a/b)] × 100,(4)

where a is the absorbance of the mixtures of the sample, linoleic acid, NH_4_SCN, and FeCl_2_ solution; and b is the absorbance of the mixtures of linoleic acid, NH_4_SCN, and FeCl_2_ solution. α-Tocopherol was used as a positive control.

#### 2.6.2. Determination of Hyaluronidase Inhibitory Activity

The inhibition against hyaluronidase was determined by measuring *N*-acetyl-glucosamine, which was the resulting product from the reaction of sodium hyaluronate and hyaluronidase [22]. The hyaluronidase enzyme activity was determined before performing each experiment, and only more than 90% enzyme activity was used in the experiment. Briefly, the samples were incubated with 15 unit/mL hyaluronidase, at the temperature of 37 °C, for 10 min, in an incubator (BMG Labtech, Offenburg, Germany). After that, 0.03 % *w/v* hyaluronic acid in phosphate buffer (pH 5.35) was added and incubated again at 37 °C, for 45 min. Then acid bovine serum albumin, made up from sodium acetate, acetic acid, and bovine serum albumin, was added into the incubate mixture to precipitate the hyaluronic acid. The absorbance of the final mixture was measured at 600 nm, using a multimode detector (BMG Labtech, Offenburg, Germany). All experiments were performed in triplicate. The inhibition of hyaluronidase was calculated by using the following equation:

% Inhibition = [1 − (a/b)] × 100,(5)

where a is the absorbance of the mixtures containing sample, hyaluronidase, hyaluronic acid, and the bovine serum albumin solution; b is the absorbance of the mixtures containing hyaluronidase, hyaluronic acid, and the bovine serum albumin solution. Ascorbic acid was used as a positive control.

#### 2.6.3. Determination of Collagenase Inhibitory Activity

The inhibition against collagenase activity was determined by measuring the decrease of substrate, FALGPA, over time [23]. The collagenase enzyme activity was determined before performing each experiment, and only more than 90% enzyme activity was used in the experiment. Briefly, the samples were incubated with 5 unit/mL collagenase for 15 min. Then 2 mM FALGPA in tricine buffer was added into the mixtures to start the reaction. The absorbance of the mixture was immediately measured after adding FALGPA solution and continuously measured for 20 min at a wavelength of 335 nm, using a multimode detector (BMG Labtech, Offenburg, Germany). All experiments were performed in triplicate. The collagenase inhibition was calculated by using the following equation:

% Inhibition = [1 − (a/b)] × 100,(6)

where a is the reaction rate of the mixtures containing the sample, collagenase, tricine buffer, and FALGPA; and b is the reaction rate of the mixtures containing collagenase, tricine buffer, and FALGPA. IC_50_ of each sample was calculated by using the program GraphPad Prism Version 2.01 (GraphPad Software, San Diego, USA). Ascorbic acid was used as a positive control.

#### 2.6.4. Determination of Elastase Inhibitory Activity

The inhibition of elastase activity was determined by measuring the product from the reaction of elastase and AAAVPN, using the spectrophotometric method described by Lee et al. [22]. The elastase enzyme activity was determined before performing each experiment, and only more than 90% enzyme activity was used in the experiment. Briefly, the samples were incubated with 4.5 unit/L elastase for 15 min. After that, 1.6 mM AAAVPN in tris HCI buffer (pH 8.0) was added into the mixtures, to start the reaction. The absorbance of the mixture was immediately measured and tracked continuously for 20 min, at a wavelength of 410 nm, using a multimode detector (BMG Labtech, Offenburg, Germany). All experiments were performed in triplicate. The inhibition of elastase was calculated by using the following equation:

% Inhibition = [1 − (a/b)] × 100,(7)

where a is the reaction rate of the mixtures containing the sample, elastase, tris HCl buffer, and AAAVPN solution; and b is the reaction rate of the mixtures containing elastase, tris HCl buffer, and AAAVPN solution. IC_50_ of each sample was calculated by using the program GraphPad Prism Version 2.01 (GraphPad Software, San Diego, USA). Ascorbic acid was used as a positive control.

### 2.7. Statistical Analysis

All data were presented as a mean ± standard deviation (SD). Statistical analysis between HO and CO was performed by the Student’s *t*-test, using the SPSS 17.0 for Windows (SPSS Inc., Chicago, IL, USA). The probability values of * *P* < 0.05, ** *P* < 0.01, and *** *P* < 0.001 were considered significant.

## 3. Results and Discussion

### 3.1. Yield and Appearance of *O. vulgare* Essential Oils

The visual appearance of *O. vulgare* essential oil from highland area was similar to that from the Mediterranean regions. The oils were a transparent yellowish liquid with the same characteristic odor. The yield, relative density, and refractive index of both oils are shown in Table 1. The results indicated that HO was of a similar quality as CO, since the relative density and refractive index, which are the parameters widely used for quality control, were exactly the same. Additionally, the properties of HO were in good agreement with a previous study of Viuda-Martos et al. [24] which reported that relative density and refractive index of *O. vulgare* essential oil obtained by steam extraction from flowers were 0.938 g/mL and 1.509, respectively. Therefore, HO which was produced from a tropical country was of a similar quality and could be used instead of CO from Mediterranean countries.

### 3.2. Chemical Compositions of *O. vulgare* Essential Oils

**Figure 1:**
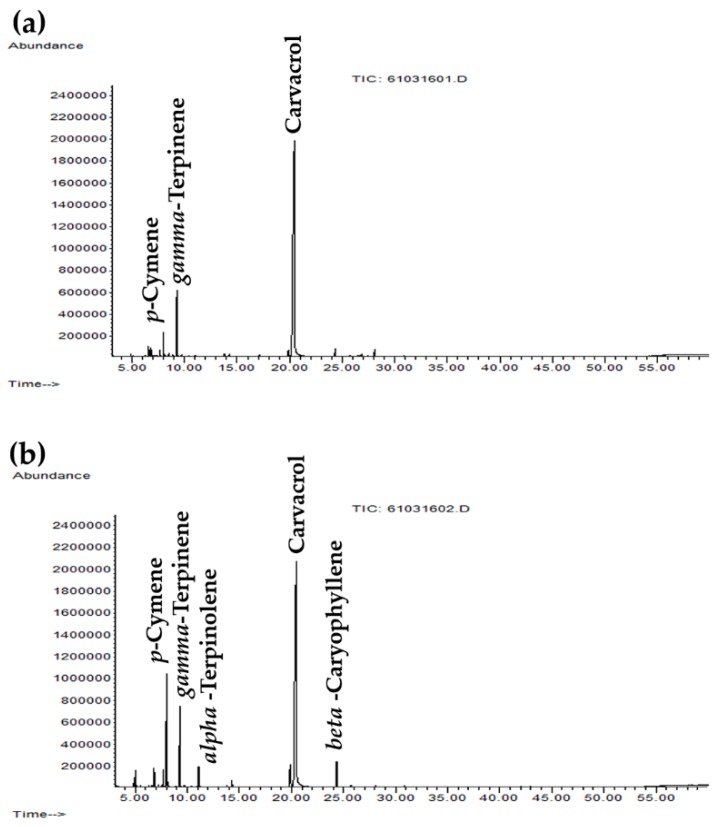
Gas chromatography–mass spectrometry (GC–MS) chromatograms of Highland *O. vulgare* oil from tropical region (**a**) and commercial *O. vulgare* from Mediterranean region (**b**).

GC–MS chromatograms of HO and CO are shown in Figure 1, and the chemical compositions of each *O. vulgare* essential oil are listed in Table 2. Carvacrol was noted as the most abundant component of both *O. vulgare* essential oils, but a higher amount was detected in HO. The differences in apparent concentrations of carvacrol were due to differences in signal-to-noise ratio of these two *O. vulgare* essential oils. Since HO contained less trace components than CO, the carvacrol content calculated as percentage was higher, although the height of GC peak of carvacrol in both oils were comparable (*p* > 0.05). Ten volatile compounds were detected in HO, accounting for 95.8% of the total oil composition, whereas thirteen volatile compounds were detected in CO, accounting for 97.2% of the total oil composition. Other than carvacrol, the volatile compounds, including *m*-thymol, *p*-cymene, and γ-terpinene, were also detected as major components in both oils. These results were in a good agreement with the previous studies that indicated carvacrol, thymol, and cymene were detected as the major components of *O. vulgare* essential oil [25,26]. Additionally, *O. vulgare* essential oil in the present study met the quality criteria specified in the Oregano monograph of the European Pharmacopoeia 01/2008:188, which was equivalent to the international standard ‘ISO 13171:2016— Essential oil of oregano’, which states that the sum of carvacrol and thymol content must be a minimum of 60% [27]. Since the sum of carvacrol and thymol contents of HO and CO were 80.7% and 68.2%, respectively, both of these oils were high quality *O. vulgare* essential oils.

Various carvacrol contents, ranging from 1.01% to 78.73%, in *O. vulgare* essential oil, have been reported in previous studies [28,29,30]. Although thymol was determined as the main compound in some cases [30,31], carvacrol amount was the major component in the present study. The results were in good accordance with several previous studies, including the study of Sivropoulou et al. [32], which revealed 79.58% of carvacrol in Greek sourced *O. vulgare* essential oil and the study of Salvo et al. [30], which revealed 26.33 ± 3.31% of carvacrol in *O. vulgare* essential oil from USA. The higher amount of carvacrol in HO (79.5%) compared to the carvacrol in CO (64.6%) and *O. vulgare* essential oils investigated in previous researches (23.43%–78.73%) should lead to superior biological activities, as carvacrol has been reported to have potent antibacterial, antifungal, antioxidant, free radical scavenging, anticholinesterase, and wound-healing activities [33,34,35,36,37,38].

**Figure 2:**
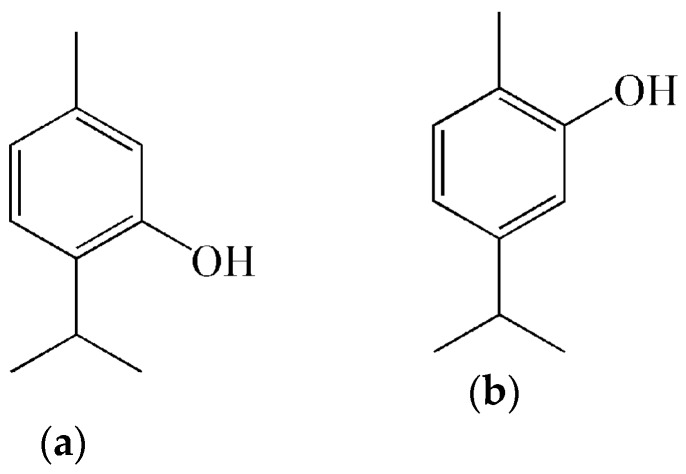
Chemical structure of thymol (**a**) and carvacrol (**b**).

Since carvacrol (5-isopropyl-2-methylphenol) is a monoterpenic phenol isomer with regards to thymol (5-methyl-2-isopropylphenol) (Figure 2), both compounds normally possess equivalent biological activities [39,40]. In some cases, thymol is a more effective and more active antioxidant than carvacrol, especially in lipid systems [41,42]. Additionally, the variability in the chemical compositions of *O. vulgare* essential oil shows a strong dependence with environment and local conditions of the plants, climatic and edaphic conditions, geographical distribution, and geographical location of the collection site [30,38,43].

### 3.3. Antioxidant Activities of *O. vulgare* Essential Oils

**Figure 3:**
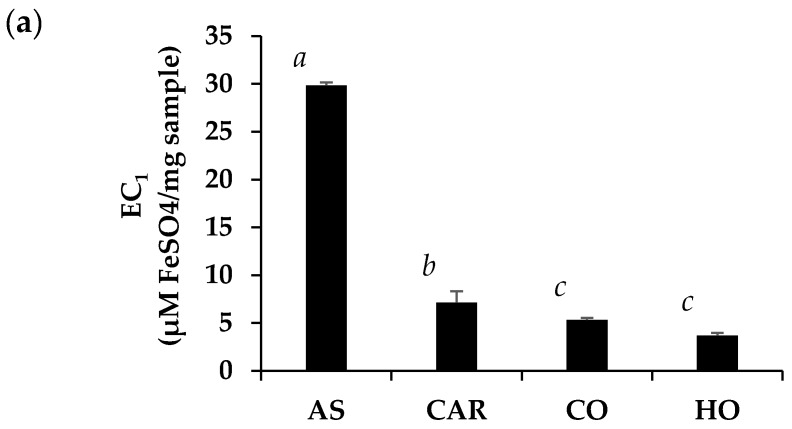

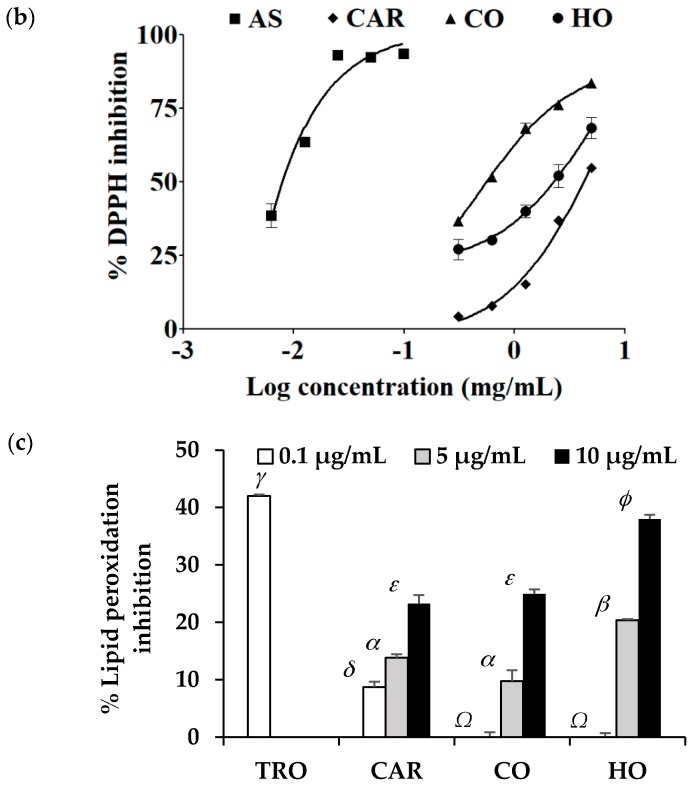
Equivalent concentration (EC_1_) (**a**), dose response curve against DPPH inhibition (**b**), and lipid peroxidation inhibition (**c**) of ascorbic acid (AS), carvacrol (CAR), commercial *O. vulgare* essential oil from Mediterranean region (CO), highland *O. vulgare* essential oil from tropical region (HO), and Trolox (TRO). The letters a, b, and c denote significantly different EC_1_ among each sample (*P* < 0.05). The symbols γ, δ, and Ω denote significantly different lipid peroxidation inhibition at the concentrations of 0.1 μg/mL (*P* < 0.05). The symbols α and β denote significantly different lipid peroxidation inhibition at the concentrations of 5 μg/mL (*P* < 0.05). The symbols ε and ϕ denote significantly different lipid peroxidation inhibition at the concentrations of 10 μg/mL (*P* < 0.05).

A collection of various antioxidant activities of *O. vulgare* essential oils is shown in Figure 3. The strong antioxidant activity of positive controls (ascorbic acid and Trolox) validated the reliability of antioxidant assays in the present study. The antioxidant activity of ascorbic acid has been mainly attributed to its reducing power (EC_1_ = 29.8 ± 1.2 µM FeSO_4_/mg sample) and radical scavenging activity (IC_50_ = 12.9 ± 0.4 µg/mL). Both O. vulgare essential oils and carvacrol had lower antioxidant activities with respect to the reference substance. Among O. vulgare essential oils, HO possessed significantly comparable reducing power to CO with the EC_1_ value of 5.3 ± 0.2 and 3.7 ± 0.3 µM FeSO_4_/mg sample, respectively (*P* > 0.05). Carvacrol was remarked as the major component responsible for the reducing power of both CO and HO because the pure carvacrol possessed significantly higher EC_1_ than that of CO and HO (*P* < 0.05). The EC_1_ value of carvacrol was 7.1 ± 0.3 µM FeSO_4_/mg sample. The results related well from the previous study, which reported that carvacrol could scavenge OH^•^ radicals at a significant level [44].

Additionally, the antioxidant results from FRAP assay related well with the DPPH assay, since the most potent preparation that could scavenge the DPPH^●^, which is a stable free radical that accepts a hydrogen radical or electron to become a stable molecule, was CO (IC_50_ = 0.6 ± 0.1 mg/mL), followed by HO (IC_50_ = 1.8 ± 0.8 mg/mL) and carvacrol (IC_50_ = 2.1 ± 0.0 mg/mL), respectively. It was noted that carvacrol did not play the major role in DPPH^●^ scavenging, since the most potent scavenger must have OH group at para position with respect to the isopropyl group on the aromatic ring [45]. Although compounds with ortho OH groups were also effective, e.g., thymol, the low content is worth noting and could not enhance the scavenging activity of the essential oils.

Although HO possessed lower reducing power than CO, HO had a comparable radical scavenging activity and significantly higher anti-lipid peroxidation efficacy than CO. Since lipid peroxidation is initiated by reactive oxygen species (ROS), which led to oxidative damages, the inhibition against lipid peroxidation would protect cells and prolong cell survival. Lipid peroxidation has been known as one of the major causes of skin ageing, since lipid peroxidation induces an increase in matrix metalloproteinase-1 (MMP-1) and matrix metalloproteinase-3 (MMP-3) [46], which in turn leads to the collagen breakdown and the appearance of skin wrinkles. To achieve the same anti-lipid peroxidation efficacy as Trolox, it was suggested that the concentration of HO needs to be 100 times higher because 10 µg/mL of HO showed comparable activities to 0.1 µg/mL of Trolox.

In the aspect of antioxidant effects, HO could be used instead of CO, since it possessed comparable radical scavenging activity to CO. Interestingly, HO possessed significantly higher lipid peroxidation inhibition than CO.

### 3.4. Anti-Skin-Ageing Activities of *O. vulgare* Essential Oils

The anti-skin-ageing activities of compounds were mainly attributed to their inhibitions against collagenase, elastase, and hyaluronidase, which were the enzymes responsible for breaking down the skin structure. Collagen fibrils, the major protein of the extracellular matrix (ECM) in dermis which strengthen the skin, can be hydrolyzed by collagenase (MMPs) and lead to skin ageing [23,47]. Similarly, elastin fibrils are other ECM components that play an important role in skin elasticity, and the cleavage of elastin fibrils by elastase leads to sagging and wrinkling skin [23,48]. Moreover, hyaluronic acid, which is a naturally occurring polysaccharide in the skin, can easily be digested by hyaluronidase and lead to the loss of skin moisture, resulting in wrinkled skin [49].

**Figure 4:**
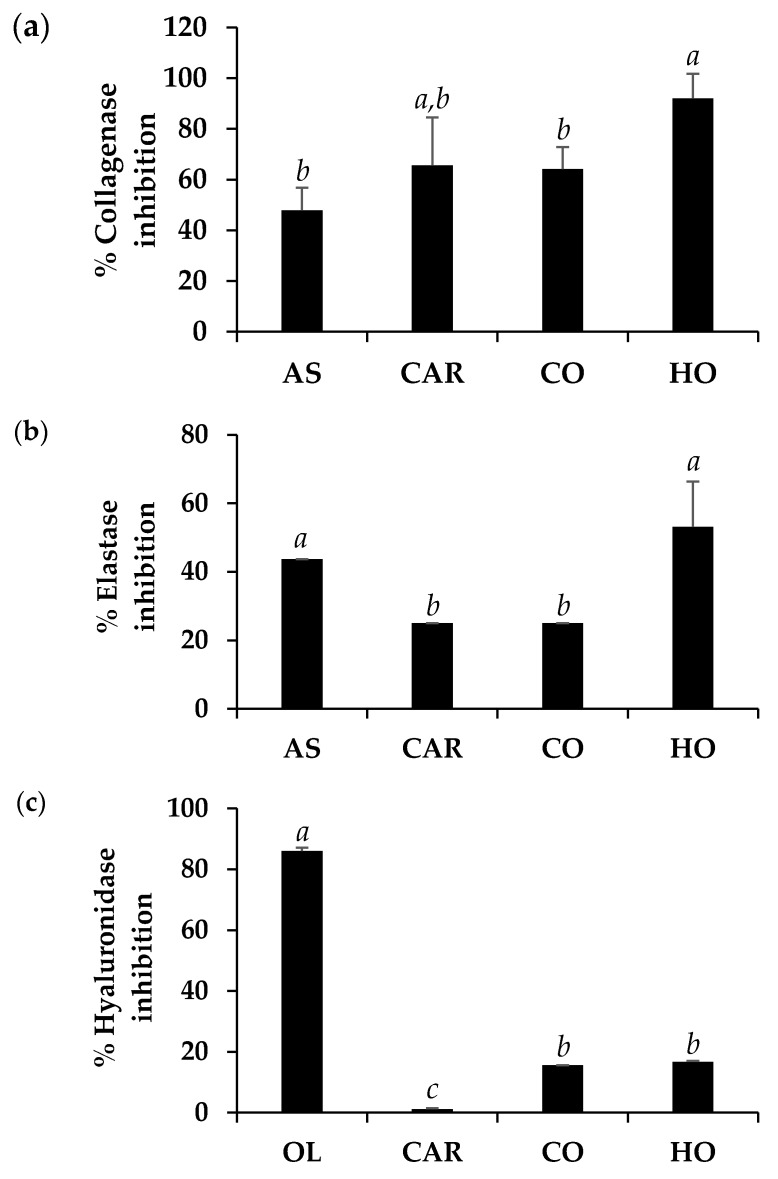
Inhibitory activity against collagenase (**a**), elastase (**b**), and hyaluronidase (**c**) of ascorbic acid (AS), oleanolic acid (OL), carvacrol (CAR), and commercial O. vulgare essential oil from Mediterranean region (CO), and highland O. vulgare essential oil from tropical region (HO). The letters, a, b, and c denoted significantly different anti-skin-ageing activity among different tested compounds (*P* < 0.05).

The anti-collagenase, anti-elastase, and anti-hyaluronidase activity of HO and CO are shown in Figure 4. Ascorbic acid was used as a positive control in anti-collagenase and anti-elastase activity determinations, since it has been reported as a potent collagenase and elastase inhibitor [50,51]. Oleanolic acid, a potent hyaluronidase inhibitor [52], was used as a positive control in the determination of anti-hyaluronidase activity.

In the present study, ascorbic acid possessed moderate anti-collagenase activity (47.9 ± 8.9%) and moderate anti-elastase activity (43.8 ± 0.0%), at final concentrations of 67 and 25 µg/mL, respectively. Surprisingly, HO possessed significant superior anti-skin-ageing activity than ascorbic acid at the same final concentration (*P* < 0.05), with the inhibitory activities against collagenase and elastase of 92.0 ± 9.7% and 53.1 ± 13.3% (Figure 4) and IC_50_ against collagenase and elastase of 35.1 ± 0.9 and 24.3 ± 0.5 µg/mL, respectively. However, carvacrol, which was a major component of HO and CO, did not seem to responsible for their anti-skin-ageing activity. The likely explanation might be due to the synergistic effect of various other compounds in CO and HO, since the activity of an essential oil did not appear to arise from a single compound [53].

On the other hand, oleanolic acid was used as a positive control since it has been reported for hyaluronidase inhibitory activity in the previous studies [54,55]. The results noted that oleanolic acid possessed a potent anti-hyaluronidase activity with inhibition of 86.0 ± 1.1%, which was much more potent than carvacrol (1.1 ± 0.3%), CO (15.5 ± 0%), and HO (16.7 ± 0.3%). Therefore, neither HO nor CO was beneficial on the retardation of hyaluronic acid loss.

The results of this study indicated that HO possessed superior anti-collagenase and anti-elastase to CO (*P* < 0.05). Therefore, HO cannot only be used instead of CO, but it showed additional anti-skin-ageing properties. The retardation of collagen and elastin loss should slow down the appearance of skin wrinkles and be beneficial for the cosmetic and cosmeceutical industries.

## 4. Conclusions

This is the first study investigating the biological activities of *O. vulgare* essential oil from highland areas of tropical countries in a comparison with the commercial essential oil from Mediterranean region in regards to skin-ageing retardation. The appearance of both *O. vulgare* essential oils was similar, and both oils were a transparent yellowish liquid with a characteristic odor. The relative density and refractive index of HO was comparable to that of CO and were 0.937 ± 0.0021 g/mL and 1.508 ± 0.0001, respectively. Carvacrol was the major component of both CO and HO but the higher amount was detected in HO (79.5%) compared to CO (64.6%). Carvacrol was identified as the major compound responsible for the antioxidant activity of both CO and HO. HO possessed significantly higher lipid peroxidation inhibition, anti-collagenase, and anti-elastase activity than CO (*P* < 0.01). Interestingly, HO possessed significant superior anti-skin-ageing activity, compared to ascorbic acid (*P* < 0.01), with collagenase, elastase, and hyaluronidase inhibition of 92.0 ± 9.7%, 53.1 ± 13.3%, and 16.7 ± 0.3%, respectively. The anti-collagenase activity of HO was superior to that of a previous reported *Ocimum sanctum* ethanolic extract of which the collagenase inhibitory activity was 77.7 ± 9.0% [48]. Therefore, HO has potential to be used as a natural active ingredient for skin-ageing retardation. Additionally, more tropical *O. vulgare* plant samples are suggested to be further investigated, since the tropical *O. vulgare* used in the present study was limited to only the highland area of Thailand.

## Figures and Tables

**Table 1:** Yield and characteristics of *O. vulgare* essential oils.

Yield and Characteristics	CO ^1^	HO ^2^
Yield (%)	N.D. ^3^	0.20 ± 0.06
Relative density (g/mL)	0.940 ± 0.0013	0.937 ± 0.0021
Refractive index	1.508 ± 0.0001	1.508 ± 0.0001

^1^ CO: commercial *O. vulgare* oil from Mediterranean region; ^2^ HO: highland *O. vulgare* oil from tropical region; ^3^ N.D.: not determined.

**Table 2:** Chemical compositions of *O. vulgare* oils. Their percentage composition and their Kovat Index (KI) values listed in order of elution.

^1^ RT (min)	^2^ KI	^3^ LKI	^4^ MW	Formula	Chemical	Amount (%)	
						^5^ HO	^6^ CO
4.9	924	924 ^a^	136	C_10_H_16_	Thujene	-	0.7
5.1	930	936 ^b^	136	C_10_H_16_	α-Pinene	-	1.2
6.8	991	991 ^a^	136	C_10_H_16_	β-Myrcene	0.9	1.7
7.2	1008	1006 ^a^	136	C_10_H_16_	α-Phellandrene	-	0.2
7.7	1028	1017 ^a^	136	C_10_H_16_	α-Terpinene	0.7	1.5
8.0	1043	1026 ^a^	134	C_10_H_14_	*p*-Cymene	2.6	10.3
8.1	1049	1030 ^b^	136	C_10_H_16_	α-Limonene	-	0.5
8.5	1068	1048 ^b^	136	C_10_H_16_	β-Ocimene	0.3	-
9.3	1101	1060 ^b^	136	C_10_H_16_	γ-Terpinene	7.6	7.6
11.1	1132	1088 ^a^	136	C_10_H_16_	α-Terpinolene	-	2.2
13.8	1177	1165 ^b^	138	C_10_H_18_	2-Bornanol	0.4	-
19.9	1309	1293 ^a^	150	C_10_H_14_O	*m*-Thymol	1.2	3.6
20.4	1322	1309 ^b^	150	C_10_H_14_O	Carvacrol	79.5	64.6
24.3	1415	1420 ^b^	204	C_15_H_24_	β-Caryophyllene	1.4	2.9
28.1	1508	1508 ^b^	204	C_15_H_24_	β-Bisabolene	1.2	0.2
					Total identified	95.8	97.2

^1^ RT: retention time; ^2^ KI: Kovat index; ^3^ LKI: Kovat index from the literature; ^4^ MW: molecular weight; ^5^ HO: highland *O. vulgare* oil from tropical region; ^6^ CO: commercial *O. vulgare* from Mediterranean region; ^a^ Kovat index on a DIM-5MS column from Bejaoui et al., 2013; ^b^ Kovat index on a DIM-5MS column from Babushok et al., 2011.
